# Mind the food: behavioural characteristics and imaging signatures of the specific handling of food objects

**DOI:** 10.1007/s00429-021-02232-9

**Published:** 2021-02-16

**Authors:** Sebastian M. Max, Philipp A. Schroeder, Jens Blechert, Katrin E. Giel, Ann-Christine Ehlis, Christian Plewnia

**Affiliations:** 1 Department of Psychiatry and Psychotherapy, Neurophysiology and Interventional Neuropsychiatry, University of Tübingen, Calwerstraße 14, 72076 Tübingen, Germany; 2 Department of Psychology, Clinical Psychology and Psychotherapy, University of Tübingen, Schleichstraße 4, 72076 Tübingen, Germany; 3 Department of Psychology, Centre for Cognitive Neuroscience, Paris-Londron-University of Salzburg, Heilbrunnerstraße 34, 5020 Salzburg, Austria; 4 Department of Psychosomatic Medicine and Psychotherapy, University Hospital Tübingen, Osianderstraße 5, 72076 Tübingen, Germany; 5 Department of Psychiatry and Psychotherapy, Psychophysiology and Optical Imaging, University Hospital Tübingen, Calwerstraße 14, 72076 Tübingen, Germany

**Keywords:** Virtual reality, Touchscreen, dlPFC, Behavioural control, Food-valuation network

## Abstract

In our world with nearly omnipresent availability of attractive and palatable high-calorie food, the struggle against overweight and obesity is a major individual and public health challenge. Preference for unhealthy food and eating-related habits have a strong influence on health, suggesting that high-calorie food triggers fast and near-automatic reaching and grasping movements. Therefore, it is important to better understand the specific neural mechanisms that control the handling of food involving a coordinated interplay between sensoric, motoric, and cognitive subsystems. To this end, 30 healthy participants (Ø BMI: 22.86 kg/m^2^; BMI range: 19–30 kg/m^2^; 23 females) were instructed to collect one of two concurrently presented objects (food vs. office tools) by manual movement in virtual reality (VR) and on a touchscreen. Parallel to the task in VR, regional brain activity was measured by functional near-infrared spectroscopy (fNIRS). In the VR and on the touchscreen, stimulus recognition and selection were faster for food than for office tools. Yet, food was collected more slowly than office tools when measured in VR. On the background of increased brain activity in the right dorsolateral prefrontal cortex (dlPFC) during food trials, this suggests more behavioural control activity during handling foods. In sum, this study emphasizes the role of the right dlPFC in faster recognition and selection of food as part of a food-valuation network, more controlled handling of food in the VR which highlights the relevance of medium for modelling food-specific embodied cognitions.

## Introduction

Rapid recognition and efficient collection of food have been pivotal skills in the successful evolutionary struggle for survival of species (Kivell et al. 2016). However, in modern environments with omnipresent availability of attractive and palatable high-calorie food, this preferential and “hard-wired” handling of food now represents a critical new challenge representing a key component in the steady rise of overweight and obesity (Spence et al. 2016). Despite widespread knowledge about the detrimental effects of excessive eating and obesity, compelling food appears capable of overcoming the rational and deliberate decision-making. A key mechanism in this connection seems to be the subjectively high value of food (Hardman et al. 2020).

Dual-system models of eating behaviour propose that an impulsive system is modulated by a reflective cognitive control system which, in the best case, supports adaptive behaviour and healthy food choice (Friese et al. 2011). Cognitive control is associated with the dorsolateral prefrontal cortex (dlPFC) (Cole and Schneider 2007; Egner and Hirsch 2005; Fales et al. 2008; MacDonald et al. 2000). The dlPFC plays a crucial role in situations with conflicts where decisions have to be made or activated representations in the working memory have to be updated (Badre and Wagner 2004). Especially the right dlPFC is involved in tasks in which response inhibition is needed to overcome impulsive prepotent actions (Blasi et al. 2006; Figner et al. 2010; Garavan et al. 2002; Knoch and Fehr 2007; Simmonds et al. 2008). Furthermore, the dlPFC is responsible for executing goal-directed behaviour based on integrating neural information of other cortical areas. As part of a specific food-valuation system, hedonic values are pre-processed in the orbitofrontal cortex (OFC) and transmitted to the dlPFC which initiates specific behaviour (Camus et al. 2009; Petrides and Pandya 1999). Already the mere presence of food stimuli can lead to an activation of the orbitofrontal cortex (Killgore et al. 2003; Morris and Dolan 2001). Attentional processes and processes of cognitive control are physiologically challenged in healthy populations if highly rewarding stimuli like food are involved (Chami et al. 2019). In different psychological paradigms, it could be shown that food can lead to either slowed (Janssen et al. 2017; Johansson et al. 2005; Nijs et al. 2010a) or speeded reactions (Castellanos et al. 2009; Hou et al. 2011; Werthmann et al. 2015), depending on the task demands and paradigms.

More complex appetitive behaviour like grasping movements can be investigated in controlled environments by means of virtual reality (VR) in combination with motion tracking. Food stimuli seem to play a specific role in grasping: Schroeder et al. (2016) reported that 3D objects of food were collected faster than ball objects in stimulus-irrelevant colour-cued grasping, but not warding. Relatively fast, but simple approach movements to various stimuli can be measured on touchscreens (Meule et al. 2019). It is quite possible that early stages of grasping behaviour reveal different behavioural patterns and that later stages are calling for a combined approach of touch- and VR-based methodologies (Gladwin et al. 2014). A systematic examination of differential and concordant effects of the medium on food valuation and behavioural control in the manual interaction with food stimuli has not been done yet and this study should provide further insights in the impact of the methodologies concerning modelling food-related differences in valuation and behavioural control.

To follow-up on the study of Schroeder et al. (2016), we established a more naturalistic scenario to investigate neurobehavioural manual movement initiation and execution based on recognition and decision processes. To this end, we implemented a binary-choice–forced-choice paradigm in a VR- and in a 2D-touchscreen setup to investigate differences in the interaction with food stimuli in natural unimanual hand movements and their neurophysiological correlates. We expect to evoke a situation where on the one hand cognitive control is challenged by presenting distractor and target stimuli simultaneously next to each other and on the other hand the food-valuation network is activated during presentation of food items. If the food-valuation network is more involved in the processing of the subjectively high value of food, we hypothesize a faster movement initiation towards food objects (Castellanos et al. 2009; Hou et al. 2011; Nijs et al. 2008, 2010b; Werthmann et al. 2013). If the cognitive control network is more involved, this should result in slower handling of food objects to prevent impulsive choices. Since the processing of alluring food stimuli likely involves more resources of the food-valuation network as well as cognitive control, it should enhance activity in the dlPFC. Accordingly, the amount of this neural activity should correlate with the interindividual differences in manual grasping movement of food objects.

## Methods

### Participants

Healthy, right-handed and non-obese participants were recruited through announcements and mails to the distributor list of a German university. In total 33, volunteers were recruited. From those, two participants were excluded due to technical problems in performing the task (bad hand tracking); one participant was excluded due to mental comorbidities. In total, 30 healthy individuals (23 women, *M*_age_ = 22.30, SD_age_ = 4.61, *N*_BMI>25_ = 8) participated in the experiment. Exclusion criteria were: left handedness, current dieting, neurological and mental diseases according to self-report, vegetarian and vegan diet, current or lifetime eating disorders according to clinical interview as well as BMI above 30. For their participation, the participants received either 8 €/h or course credits. The study was approved by the ethics committee of the Medical Faculty Tübingen (829/2018BO2) and all participants gave informed consent.

### Apparatus

#### Virtual reality (VR)

During the behavioural task, participants were seated in a comfortable chair and wore a head-mounted display (HMD) which allowed for continuous tracking of head rotation (Oculus Rift CV1; Oculus VR, Inc., Menlo Park, USA). The HMD consists of two screens, both with a resolution of 1080 × 1200 pixels. The inter-pupillary distance was adjusted for each participant individually. A near-infrared sensor (Leap Motion Inc., San Francisco, USA) tracked the trajectories of the participants’ hand. These trajectories were streamed in real time into the stereoscopic display so the participants could interact with virtual stimuli through actual movements of their hand, comparable to our previous setups (Lohmann et al. 2018; Schroeder et al. 2016). Stereoscopic presentation was controlled by Unity 3D (5.6.2f1) with a bundled version of OVRPlugin. The leap motion device was positioned on a small table in front of participants and allowed for object interactions with the dominant hand in an area of approximately 1600 cm^2^ (effectively covering most of the grasping range of the participants). The 3D-stimuli originated from the asset-store of Unity (Unity Technologies, San Francisco, USA) or Blender-models (Blend Swap, LLC). To realize matching of shape and colour of the stimuli, the objects got rescaled and recoloured. The stimuli set consists of the variations originating from the subcategories (balls, food, office objects). Ledoux et al. (2013) could show that food stimuli in the VR are comparable to pictures of food and real-life food concerning triggering food craving. In total, 48 different stimuli were used. Stimuli were rated by the participants concerning valence, arousal, grasp to urge, aesthetics, subjective estimated size and comfort of grasping on a continuous scale ranging from 0 to 100. Overall, stimuli were comparable regarding several practical dimensions except consumption value, see Appendix A.

#### 2D touchscreen

To operate on the 23-inch 2D touchscreen (iiyama ProLite T2336MSC) with a resolution of 1920 × 1080 pixels, the participants wore touchscreen gloves. The start position of the hand was marked by a hand symbol on the screen. The location of the hand symbol was in the bottom-mid of the touchscreen that was positioned in a landscape orientation. The distractor and target stimuli were presented in the top left and top right corner of the horizontally oriented display. The resolution of the stimuli was 150 × 150 pixels. Most of the stimuli originated from a public picture data base (Blechert et al. 2014), which were used in a previous experimental setup (Meule et al. 2019). To realize matched photorealistic stimuli for the VR stimuli, the rest of the photorealistic stimuli were downloaded from the internet. Ledoux et al. (2013) could show that food-pictures are comparable to real-life food concerning triggering food craving. Half of the stimuli consisted of screenshots of the VR stimuli, the other half of photorealistic stimuli. Stimuli were rated by the participants concerning valence, arousal, grasp to urge, aesthetics, subjective estimated size and comfort of grasping on a continuous scale ranging from 0 to 100. Overall, stimuli were comparable regarding several practical dimensions except consumption value, see Appendix B.

#### Functional near-infrared spectroscopy (fNIRS)

An ETG-4000 Continuous Wave Optical Topography System (Hitachi Medical Co., Japan) was used to measure relative oxygenated (O_2_Hb) and deoxygenated (HHb) blood concentration as indicators for brain activity (Fallgatter et al. 2004). The sampling rate was 10 Hz. Two 3 × 3 probe-sets with 12 measurement channels each and an inter-optode distance of 30 mm were placed over the left and right prefrontal cortex after the HMD was mounted. According to the international 10–20-system, one probe-set was placed over F3 (channel #7) whereas the other probe-set was placed over F4 (channel #19; see Fig. 4) (Jasper 1958). Using this configuration, the NIRS channels were predominantly located over the left and right dorsolateral (Brodmann areas 9 and 46) and inferior frontal cortex (Brodmann areas 44 and 45), as extrapolated from reference points based on the Colin 27 template (Singh et al. 2005; Tsuzuki and Dan 2014; Tsuzuki et al. 2007).

### Procedure

Participants were instructed to not eat at least 3 h before the experiment and were asked at the appointment if they actually didn’t eat. All participants declared conformity with this instruction. The whole experiment was conducted in a single session lasting approximately 2 h and typically took place between 11.00–13.30 and 15.30–19.30. After assessing demographic data, the absence of a current and lifetime eating disorder was examined by the section H of the SCID-I (Wittchen et al. 1997). Thereafter, participants were weighed on a scale. Height was determined by self-report. Food craving assessed by the Food Craving Questionnaire–State (Meule et al. 2012) before and after the task in the VR and can be looked up in Appendix C.

**Fig. 1:**
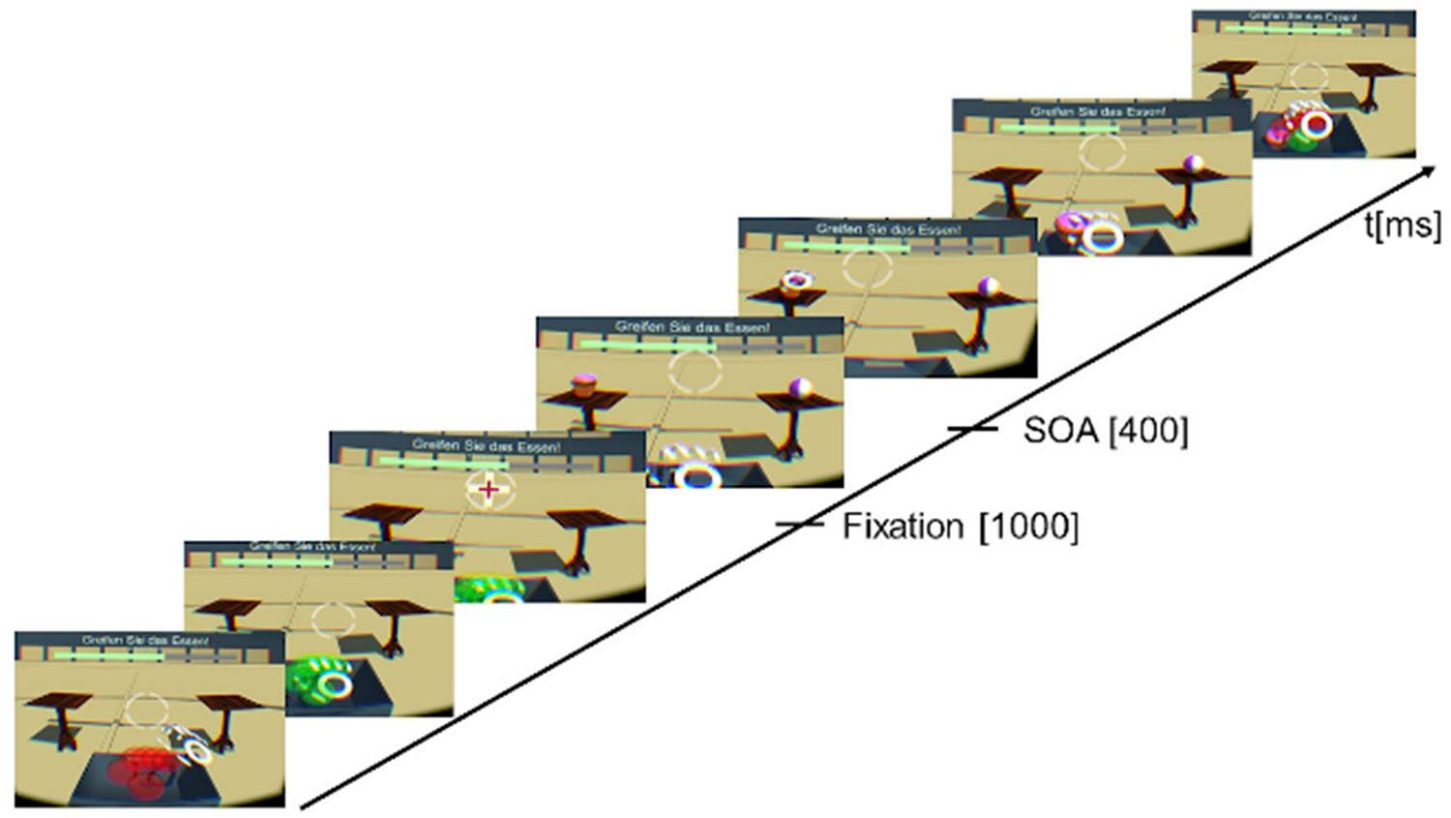
An exemplary trial of the condition “food”. The target stimulus which had to be grasped was a chocolate cupcake with pink icing. After the initial hand pose matches with the standardized hand pose, a fixation cross had to be aligned with the crosshair of the HMD for one second. With a stimulus asynchrony of 400 ms, the target and distractor stimulus were presented on the left and right table

Afterwards, participants were equipped with the HMD and the behavioural task in the VR started. The task started with practice trials to familiarize the participant with operating the system. Then the fNIRS probe-set was mounted on the participants’ head above the HMD. To start a trial, the participants had to put the hand in a standardized position. The start position of the right hand was marked by seven red coloured spheres which turned green if the start position was right. Subsequently, a fixation cross was presented which had to be aligned with a crosshair of the HMD for a duration of one second. Due to this fixation the participants had a standardized start position for their head and their gaze. With a stimulus onset asynchrony of 400 ms, a target and a distractor stimulus were presented concurrently next to each other. The participants were instructed not to move their hand until the stimuli were presented, otherwise an error message was displayed. If the target stimulus was presented on the left table, the distractor stimulus was presented on the right table and vice versa. The positions of target and distractor stimuli were counterbalanced within a block. Each block consisted of 32 trials. The trial was finished, when the target stimulus was grasped and placed inside the box. If the participants placed the target stimulus outside of the box, grasped the wrong stimulus or took longer than four seconds for the grasping, an error message was displayed and the next trial started. The distance between the start position and the target stimulus was around 40 cm. The whole behavioural task in the VR consisted of six blocks. Across participants, block order was counterbalanced and each participant was randomly assigned to a block order. After each block, there was a short self-paced break. The whole task took about 15 min. An exemplary trial is shown in Fig. 1. Afterwards, the behavioural task was conducted at the 2D touchscreen without fNIRS.

The participants were seated in front of the horizontal oriented touchscreen with an angle of approximately 15° relative to the tabletop. The 2D touchscreen task was analogous to the task in the VR with the identical block order as in the VR. At the start of each trial, the participant had to touch a hand icon on the display with five fingers. After 500 ms, a fixation cross was presented for 1 s and with a stimulus asynchrony of 400 ms, the target stimulus and distractor stimulus were presented concurrently on the left and right top corner of the display.

**Fig. 2:**
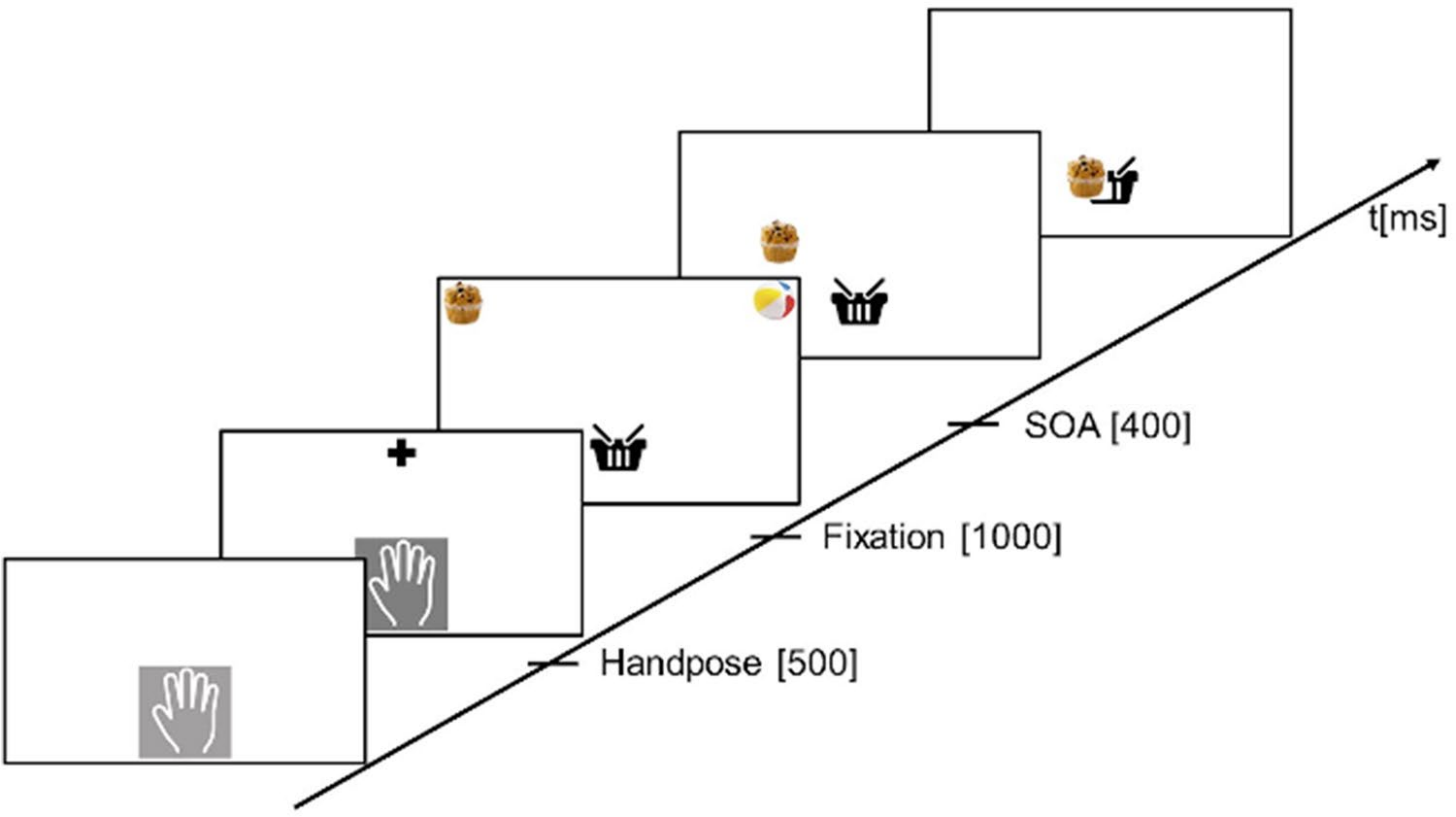
An exemplary trial of the condition “food”. The target stimulus which had to be grasped was a chocolate cupcake. After the hand icon has been touched with five fingers for 500 ms, a fixation cross was presented for one second. With a stimulus asynchrony of 400 ms the target and distractor stimulus were presented on the left and right top corner of the touchscreen

According to the block instruction which was given before each block, the participant had to collect the target stimuli and drag it into a 2D model of a box. Due to faster startup procedures of each trial and faster movements in general, the whole task at the touchscreen took only around 10 min. An exemplary trial is shown in Fig. 2.

In both the VR task and the 2D-touchscreen task, each of the six blocks consisted of 32 trials. In two blocks each there were instructions to grasp different stimulus categories as fast and as correct as possible: food, balls or office tools. In each block, only two stimulus categories were presented. The order of the instruction was counterbalanced across all participants. Each stimulus category was twice selected as target and twice as distractor stimulus and paired with each other counterbalanced. Each variation of a stimulus category was presented randomized and balanced on the left and right table. Each pairing of the stimulus subcategories was realized.

### Data analysis and statistics

#### Object rating analysis

All statistical inferences are conducted on a significance level of 5%. To estimate significance of valence of the different object categories (food, office tools, balls) paired *t* tests are conducted. Exploratory, to investigate the correlation between subjective food valence rating and reaction times, Pearson’s correlation tests were conducted.

#### Behavioural data analysis

All statistical inferences are conducted on a significance level of 5%. Incorrect trials were excluded from all analyses (2.52%). Reaction times above 2000 ms and below 200 ms for the movement onset as well as reaction times above 4000 ms for the collection time on the touchscreen and in the VR were considered premature/incorrect. Movement onset is defined as the time between the presentation of the objects and the movement onset. Collection time is the time span after the participant’s hand has left the start position until the participant’s hand with the critical target has reached the position above the collection box where the target gets dragged into. Further, values deviating more than 2.5 SD, from individual cell means were considered outlier responses. 2.78 percent of the trials in the VR and 2.98 percent of the trials at the touchscreen were excluded. Only reaction times were analyzed as the block design is expected to affect reaction times rather than error rates (Zeligman and Zivotofsky 2017). To account for individual differences in motor grasping of grasp-affordant objects, we decided to standardize the reaction times for the food and office objects in relation to the ball objects. As grasping ball objects resembles exclusively motor actions which have to be executed fast and in an automatic manner, this seems to be a valid baseline correction. Furthermore, the ball objects are less complex than the other objects and demand less cognitive functions like planning. Each trial was subtracted by the individual’s mean reaction time for ball objects. To measure effects of the different target stimuli on the manual grasping times in the VR compared to the touchscreen, the linear mixed model approach was used to account for individual differences in grasping food stimuli. All linear mixed models were calculated by the *lme4-*package of R (Bates et al. 2014). The linear mixed effect model approach contains a random effect for each subject which comprises the interindividual differences to the manipulated fixed effects, whereas the fixed effects are the averaged prediction of the fixed effects on the reaction times across all participants. To estimate the significance of each fixed effect log-likelihood tests between a linear mixed model with the fixed effect and a linear mixed model without the fixed effect were conducted. Two fixed effects were tested within the linear mixed model: the medium (VR vs. Touchscreen) and the category of the target stimuli (Food vs. Balls). Post hoc tests to test the different contrasts within a fixed effect of the linear mixed model, the *lsmeans*-package of R was used. This is based on the method of the least-squares means and post hoc tests are adjusted by Tukey method (Lenth 2016). To estimate the degrees of freedom of the post hoc tests, the Satterthwaite formula for degrees of freedom was used. Effect sizes for fixed effects were estimated by *f*^2^ which can also be used in mixed linear models (Aiken et al. 1991; Lorah 2018).

An individual bias separately for movement onset and collection time for each participant was calculated by the difference of the ball-standardized reaction times of food objects and the ball-standardized reaction times of office tools:

(RT_Office_ − RT_Ball_) − (RT_Food_ − RT_Ball_). Therefore, a higher value indicates a more prominent shift towards food.

Further exploratory analysis with linear mixed models was carried out to investigate the impact of the valence differences of the two different objects that the participants faced during each trial on the reaction times towards food. For this, reaction times were aggregated across the participants. The difference score served as a fixed effect and a random intercept on each target stimulus was modelled.

#### fNIRS data analysis

Concentration changes of oxygenated (O2Hb) and deoxygenated haemoglobin (HHb) concentration were used for the fNIRS analysis, which was conducted using customized Matlab scripts (Matlab 2017a; The MathWorks, Inc., Natick, MA, USA). First, missing channels were interpolated before motion-based artefacts were corrected by the temporal derivative distribution repair (TDDR) method (Fishburn et al. 2019). To minimize further artefacts of non-neural causes, signal improvement relying on the assumption of a negative correlation between oxygenated and deoxygenated haemoglobin was conducted (Cui et al. 2010) during which the two signals were combined to one “true oxy signal” which was then further analysed. A bandpass filter of 0.01 to 0.10 Hz was applied and around 3.60 percent of the channels with remaining artefacts were interpolated manually. A Gaussian kernel filter with a standard deviation of *σ* = 40 was then used to remove global physiological artefacts (e.g., related to respiration) (Zhang et al. 2016). Finally, the data were z-standardized and the mean z-transformed amplitude ranging from 10 to 40 s following the start of the block was individually exported for further statistical analysis [with a pre-task baseline of 5 s resting and separately for the individual average of both “food blocks” (food as target with distractor of balls or office tools), both “ball blocks” (ball as target with distractor of food or office tools) and both “office blocks” (office tools as target with distractor of food or ball)].

To be in line with previous statistical analysis, we decided to standardize the fNIRS BOLD response for the food and office objects in relation to the ball objects. The analysis focusses on the ball-object-corrected fNIRS BOLD signal between food and office objects. To measure effects of the different target stimuli on the fNIRS BOLD response, paired *t* tests between the conditions were performed and Bonferroni-corrected for multiple comparisons. Those paired *t* test were performed on the channels covering Brodmann area 9 (channel number (Ch) 8, 11, 13, 15, 16, 18, 20) and 46 (Ch 5, 9, 19, 21, 22) which both contribute to the dorsolateral prefrontal cortex (dlPFC), as well as Brodmann area 44 (Ch 6, 23) and 45 (Ch 2, 4, 7, 24) which both contribute to the inferior frontal gyrus (IFG).

Correlations between the fNIRS BOLD response and the individual biases of the manual actions were also calculated by Pearson’s correlation test. An fNIRS BOLD response bias was calculated by the following formula:

(fNIRS-BOLD_Food_ − fNIRS-BOLD_Ball_) − (fNIRS-BOLD_Office_ − fNIRS-BOLD_Ball_). Therefore, a higher value indicates a higher shift towards food.

The individual behaviour biases for each participant were correlated with the individual fNIRS-BOLD response bias in the VR solely as there was no concurrent fNIRS measure at the touchscreen. Target areas of the fNIRS BOLD response were Brodmann area 9, 46, 44 and 45.

## Results

### Object ratings

In the VR, a significantly higher mean score concerning valence is reported for food items (*M*_VR_ = 66.92, SD_VR_ = 14.68) than for office tools (*M*_VR_ = 51.29*, *SD_VR_ = 12.65), *t*(29) = 4.62,* p* < 0.001, and for balls (*M*_VR_ = 58.06*, *SD_VR_ = 10.97). At the touchscreen, the mean valence of food items (*M*_TS_ = 71.48, SD_TS_ = 13.74) is significantly higher than for office tools *(M*_TS_ = 51.15, SD_TS_ = 12.87), *t*(29) = 6.67, *p* < 0.001, and significantly higher than for balls (*M*_TS_ = 58.75, SD_TS_ = 13.66), *t*(29) = 4.78, *p* < 0.001.

### Effects of food on different stages of manual movement in VR and touchscreen

#### Movement onset

Including the category of target stimuli as a fixed effect in the mixed model leads to a significantly better model than the random intercept-only model, χ^2^(1) = 106.87, *p* < 0.001, *R*^2^ = 0.09. The movement onset for the food objects as a target was significantly faster than for the office objects (*M* = 19.04 ms), *t* = 10.38, *SE* = 1.84*, p* < 0.001, *f*^*2*^ = 0.016.

Including the medium as fixed effects in the mixed model leads to a significantly better model than the random intercept-only model, χ^2^(1) = 355.49, *p* < 0.001, R^2^ = 0.12. The movement onset for the objects in the VR is significantly slower than at the touchscreen (*M* = 34.43 ms), *t* = 19.18, *SE* = 1.80, *p* < 0.001, *f*^*2*^ = 0.050.

**Fig. 3:**
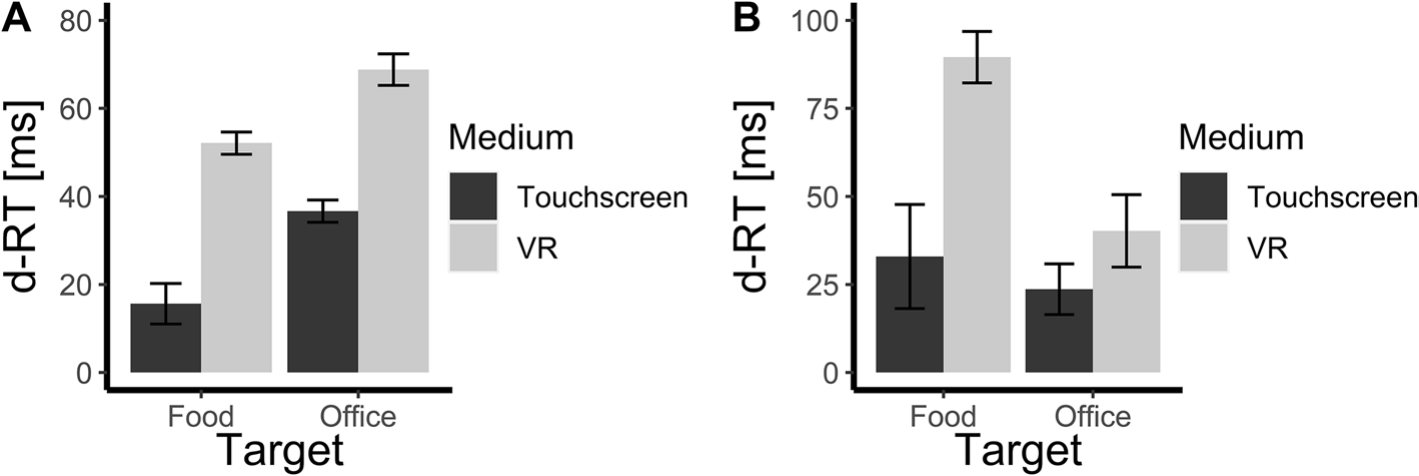
Movement onset (**a**) and collection time (**b**) in relation to the movement onset reaction times of the ball objects. The bars represent the standard error of fixed effect estimates. d-RT were calculated by subtracting the reaction time of the target stimulus from the reaction time of the ball objects

Including an interaction between the two fixed effects does not lead to a significantly better model, χ^2^(1) = 1.49, *p* = 0.222, R^2^ = 0.14. This means the different levels of the fixed effects do not interact with each other. In Fig. 3a the ball-object-corrected reaction times of the movement onset are depicted. For the raw reaction times of the movement onset, see Appendix D.

#### Collection time

Including the category of target stimuli as fixed effects in the mixed model leads to a significantly better model than the random intercept-only model, χ^2^(1) = 30.58, *p* < 0.001,

R^2^ = 0.11. The same applies to the medium. This fixed effect in the mixed model leads to a significantly better model than the random intercept-only model, χ^2^(1) = 49.27, *p* < 0.001,

R^2^ = 0.11. Furthermore, those two fixed effects interact, χ^2^(1) = 15.11, *p* < 0.001, R^2^ = 0.12. Whereas the collection time of food and office objects does not differ significantly at the touchscreen (*M* = 9.31 ms), *t* = − 1.29, SE = 7.19, *p* = 0.566, the collection time of office objects is significantly faster than the collection time of food objects in the VR (*M* = 49.34 ms), *t* = − 6.70, SE = 7.36, *p* < 0.001, *f*^*2*^ = 0.002. That means, as soon as the participants have left their initial hand position, they collect office objects quicker than food objects, but only in the VR. In Fig. 3b the ball-object-corrected reaction times of the collection time are depicted. For the raw reaction times of the collection time, see Appendix D.

#### Object ratings and manual action in VR and at the touchscreen

In the VR, neither the movement onset time correlate with valence of food items (*r* = 0.18), *t*(14) = 0.67, *p* = 0.512, nor the collection time (*r* = 0.22), *t*(14) = 0.86, *p* = 0.405. At the touchscreen, neither the movement onset time correlates with valence of food items (*r* = − 0.11), *t*(14) = − 0.42, *p* = 0.680, nor the collection time (*r* = 0.30), *t*(14) = 1.16, *p* = 0.266.

For the movement onset in the VR, there is a significant impact of the difference of the valence between the two objects the participant faced during each trial on the movement onset (*β* = − 1.45, *p* < 0.001). For each rating point difference concerning subjective valence between the two-faced objects, the movement onset for the food object got 1.45 ms faster. For the collection time in the VR, the difference of the valence is not a significant predictor (*β* = 0.24, *p* < 0.816). At the touchscreen, the difference of the valence does not predict the movement onset (*β* = − 0.56, *p* = 0.056), nor the collection time (*β* = − 1.33, *p* = 0.146).

### Functional near-infrared spectroscopy (fNIRS)

#### Effects of stimuli categories on fNIRS BOLD response in VR

**Fig. 4:**
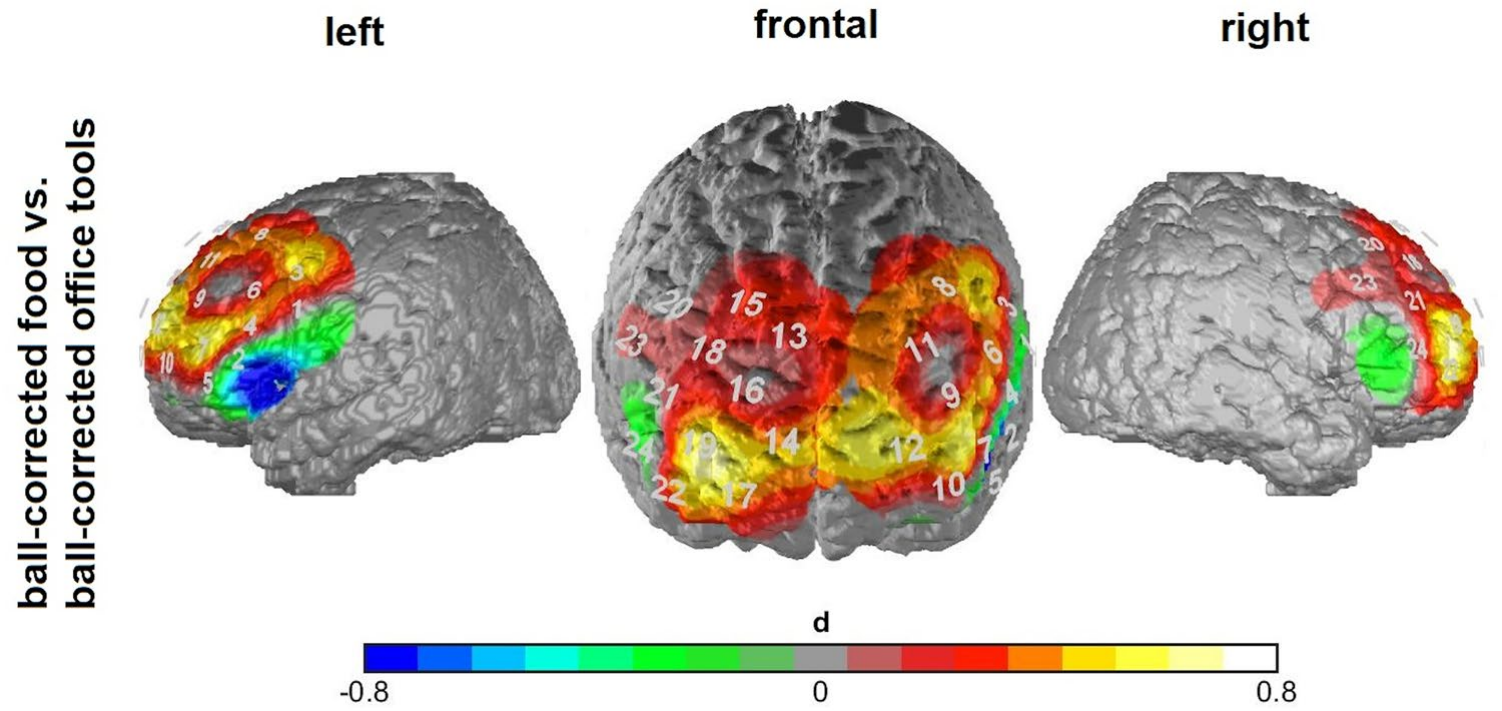
Map for the functional near-infrared spectroscopy (fNIRS) data showing neural activity during the VR task. The numbers depicted resemble the corresponding channel numbers. The contrast between the ball-corrected food condition and the ball-corrected office condition is depicted and effect sizes are reported. Positive values indicate activation, negative values indicate deactivation

A strong prefrontal activation in the ball-standardized food condition in contrast to the ball-standardized office condition can be seen (Fig. 4). Especially in the region of the right dlPFC (Ch 19), a significantly higher activation in the food condition can be observed, *t*(29) = 3.29, *p* = 0.047. All other comparisons of the two standardized condition in other brain regions are not significant. All paired t tests conducted on the channels covering dlPFC and IFG are listed in Appendix E.

#### fNIRS BOLD response bias and manual movement in VR

**Fig. 5:**
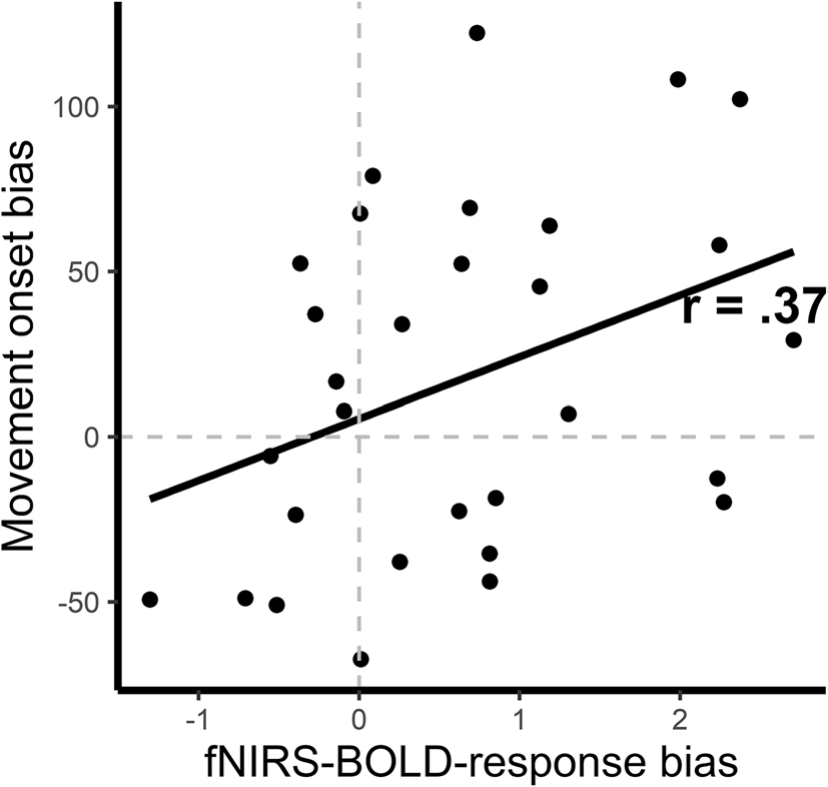
Correlation between individual movement onset bias and fNIRS BOLD response bias in the right dlPFC in the VR. Movement onset bias was calculated by subtracting the ball-standardized reaction times for food objects from the standardized reaction times for office tools. A positive value indicates faster reaction times to food stimuli compared to office stimuli. fNIRS BOLD response bias was calculated by subtracting the ball-standardized z-transformed fNIRS BOLD response for office tools from the ball-standardized z-transformed fNIRS BOLD response for food objects. A positive value indicates more neural activity for food compared to office tools

A significant positive correlation (*r* = 0.37) between the fNIRS BOLD response bias of the right dlPFC and the movement onset bias is observed, *t*(28) = 2.10, *p* = 0.044. The stronger the fNIRS BOLD response to food objects in relation to office objects, the faster the decision to lift off the hand and to start approaching the food object compared to the office object. This correlation is depicted in Fig. 5. The correlation (*r* = 0.13) between the fNIRS BOLD response bias of the right dlPFC and the collection time bias is not significant, *t*(28) = 0.71, *p* = 0.486.

## Discussion

The present study revealed attentional and behavioural processes during the handling of food objects by assessing recognition and reaction times in both a VR setup and on a touchscreen device while further assessing fNIRS based brain activity during VR. We found that manual interaction with food stimuli goes along with enhanced neural activity in the right dlPFC. In line with our hypothesis concerning the involvement of a food-valuation network, we found a faster movement onset towards food as compared to office tools in both interfaces (VR and touchscreen) accompanied by higher neural activity in the right dlPFC. In line with our hypothesis concerning cognitive control, after movement initiation, a slower handling of food compared to office tools was observed in the VR. Of note, this effect was absent on the touchscreen.

A pivotal finding of this study is the enhanced neural activity in the right dlPFC when food stimuli were the target for the grasping movement. This food-related activity in the right dlPFC is in line with the proposal of the right dlPFC as a part of a specific food-valuation system (Camus et al. 2009). This model assumes an interconnection of the dlPFC and the orbitofrontal cortex (OFC) (Petrides and Pandya 1999). Hedonic values of stimuli are processed in the OFC and transmitted to the dlPFC to execute specific goal-dependent behaviour (Camus et al. 2009). Even if the participants are not free to choose which object they want to collect, an activation of a specific food-valuation system in the right dlPFC can be assumed since an adjustment of behaviour is required in each trial according to the localization of the food object. Apparently, food captures more neural resources in, for instance, estimating stimuli specific values and establishing goal-dependent behaviour. Therefore, it is reasonable to assume an additional food-specific effort.

The speeded reaction times of the first stage of manual interaction, the initiation of movement are in line with previous research. This stage involves the recognition and selection of two different object categories. Research using the visual dot probe paradigm showed elevated attentional processes towards food stimuli when compared to control stimuli (Castellanos et al. 2009; Hou et al. 2011; Nijs et al. 2008, 2010b; Werthmann et al. 2013). This could be due to the significantly higher value and survival relevance of food compared to office tools. Even if we did not find a significant correlation between valence of food objects and the reaction times, we could find a significant impact of the differences concerning subjective valence of the two concurrently presented stimuli. A higher difference in subjective valence led to faster movement initiation, thus highlighting the role of value in attentional selection and movement initiation processes involving food. On a neuropsychological level, those elevated attentional processes are associated with the right dlPFC, a brain region which is mostly known for its role in response inhibition (Blasi et al. 2006; Mostofsky and Simmonds 2008). The neuropsychological correlates of the current study highlight a more prominent role of the right dlPFC in selecting different target stimuli rather than suppressing an automatic response: the higher the brain activity in the right dlPFC, the faster the manual movement initiation. This correlational finding qualifies the role of the dlPFC in regulating early goal-directed behaviour in the interaction with food objects (Cornier et al. 2010; Horstmann et al. 2011).

The second stage of manual interaction, the collection time, was slower in food than in office tools and therefore is in line with the hypothesis of a cognitive control network. In contrast to the previous study by Schroeder et al. (2016) reporting a speeded collection time of food, we implemented a new paradigm to assess more naturalistic behaviour. In the current study, the participants had to react to a goal-relevant feature of this task and explicitly discriminate and select one out of two concurrently presented stimulus categories. Accordingly, a more conscious and controlled handling of food could indicate the higher hedonic value of food and its associated need to suppress an impulsive behaviour. As there was no significant correlation between the collection time and the brain activity in the right dlPFC, the involvement of the right dlPFC as a cognitive control network was not as prominent as part of a food-valuation network. Alternatively, attentional biases can be influenced by both appetitive and aversive motivational processes (Field et al. 2016). The finding that differences in manual interaction with food were more prominent in the VR than at the touchscreen highlights the crucial role of response medium and the need for more methodological research. These differences in interaction with food stimuli provide information about its underlying mechanisms of food-valuation and cognitive control. It can be assumed that, particularly in the VR involving more complex and naturalistic behaviour and a higher level of immersion, a more careful and thus slower handling of food is a consequence of higher significance or personal importance of food objects. Consequently, VR seems more promising in possible future applications to modify specific behavioural biases, for example in eating disordered samples, whereas touchscreens potentially allow a wider and easier dissemination. While both media are able to reflect food-specific cognitive differences in the recognition-phase, VR seems to be more sensitive in modelling embodied cognition in the specific handling of food.

## Conclusion

In sum, by means of a food-decision task in VR, we were able to document differences in interaction speed with food stimuli partially linked with higher activation in the right dlPFC. Faster movement initiation on the one side and slower handling of food on the other is consistent with the relatively higher appeal of food objects and a correspondingly more controlled interaction. These findings underline the significance of food valuation and cognitive control networks in manual interactions with food and the particular role of the right dorsolateral prefrontal cortex. Food-specific behaviour was more evident in the VR than at the two-dimensional touchscreen, which emphasizes the relevance of medium in modelling food-specific behavioural differences.

Investigating dynamics in a sample with disinhibited eating behaviour like obese subjects or patients with binge-eating-disorder could offer further insights in relevant behavioural characteristics and their neurophysiological signatures. These findings support the notion that targeted network stimulation and bias retraining may provide promising perspectives for an individualized modulation of disorders associated with an increased food bias. In this context, the use of immersive VR technology seems to be most promising for inducing behaviourally relevant effects.

## Data availability statement

The data that support the findings of this study are available from the corresponding author upon reasonable request.

## Data Availability

Not applicable.

## Appendix A Ratings of VR stimuli

**Table:**

Stimulus	Mean valence (SD)	Mean arousal (SD)	Mean urge to grasp (SD)	Mean aesthetics (SD)	Mean subjective size (SD)	Mean grasp comfort (SD)
Baseball	57.69 (13.28)	42.89 (19.27)	47.41 (19.33)	52.13 (17.06)	42.57 (17.71)	64.87 (18.36)
Baseball_1	65.23 (14.98)	42.33 (26.15)	50.10 (22.72)	65.00 (18.46)	45.33 (18.12)	71.10 (22.79)
Baseball_2	48.20 (16.89)	47.10 (24.60)	44.30 (20.81)	40.73 (22.04)	42.13 (18.80)	61.57 (20.83)
Baseball_3	54.23 (16.93)	42.33 (22.20)	44.03 (23.88)	49.50 (20.86)	39.97 (18.98)	62.63 (21.15)
Baseball_4	63.10 (19.30)	39.80 (20.20)	51.20 (22.93)	53.27 (22.22)	42.83 (19.15)	64.17 (21.12)
Beachball	56.78 (14.14)	46.27 (17.79)	47.81 (18.22)	50.86 (18.08)	61.93 (13.35)	64.17 (15.93)
Beachball_1	60.60 (17.04)	51.13 (20.11)	49.80 (21.57)	55.47 (19.15)	61.17 (15.73)	62.83 (16.97)
Beachball_2	58.70 (15.92)	46.93 (20.31)	50.83 (18.20)	50.20 (22.11)	60.50 (13.71)	66.03 (17.48)
Beachball_3	53.57 (16.89)	44.33 (21.67)	45.13 (22.76)	48.83 (25.12)	63.10 (16.59)	62.40 (18.16)
Beachball_4	54.27 (16.42)	42.67 (21.77)	45.47 (20.02)	48.93 (19.63)	62.93 (14.15)	65.40 (19.61)
Handball	62.03 (17.77)	54.92 (19.61)	53.35 (18.77)	55.45 (19.87)	66.93 (14.82)	69.28 (17.54)
Handball_1	63.37 (22.40)	55.87 (23.69)	53.70 (25.03)	58.37 (26.62)	67.40 (15.09)	70.80 (18.89)
Handball_2	61.90 (23.67)	53.33 (26.67)	51.87 (22.85)	46.00 (28.60)	68.30 (15.53)	67.23 (22.99)
Handball_3	58.97 (20.93)	54.30 (20.76)	53.43 (21.96)	56.10 (27.08)	66.03 (15.31)	68.77 (15.84)
Handball_4	63.87 (18.45)	56.17 (22.92)	54.40 (19.18)	61.33 (20.16)	65.97 (16.62)	70.33 (17.46)
Tennisball	55.73 (11.17)	47.84 (18.35)	50.57 (18.08)	51.45 (17.15)	41.98 (19.82)	65.41 (16.00)
Tennisball_1	50.93 (15.80)	44.77 (20.02)	49.23 (23.54)	47.07 (22.98)	42.10 (20.72)	63.70 (20.52)
Tennisball_2	57.60 (14.45)	44.60 (20.71)	49.40 (17.34)	57.37 (21.48)	43.67 (21.40)	68.30 (18.26)
Tennisball_3	54.97 (17.94)	51.33 (21.96)	50.77 (21.15)	49.87 (21.90)	41.43 (20.82)	62.03 (22.33)
Tennisball_4	59.40 (19.05)	50.67 (22.50)	52.87 (22.98)	51.50 (26.60)	40.70 (19.13)	67.60 (16.91)
Burger	64.09 (18.16)	64.30 (17.66)	62.93 (19.35)	52.15 (20.19)	65.97 (11.49)	69.28 (17.64)
Burger_1	62.87 (21.15)	65.33 (21.28)	64.50 (21.22)	49.53 (25.27)	60.73 (13.21)	66.87 (19.43)
Burger_2	71.40 (20.99)	62.57 (20.50)	66.87 (21.17)	60.27 (22.72)	63.17 (14.73)	71.27 (20.43)
Burger_3	69.03 (22.17)	62.97 (23.42)	63.50 (23.63)	60.70 (24.83)	69.40 (14.67)	70.47 (17.80)
Burger_4	53.07 (24.53)	66.33 (22.15)	56.83 (25.41)	38.10 (24.45)	70.57 (14.75)	68.50 (20.28)
Cupcake	67.98 (15.99)	64.91 (16.48)	59.17 (20.22)	63.18 (16.81)	45.31 (16.01)	68.64 (16.88)
Cupcake_1	75.53 (15.33)	68.10 (19.21)	62.83 (25.73)	74.30 (16.28)	46.40 (19.12)	70.80 (19.80)
Cupcake_2	66.77 (15.63)	58.37 (22.02)	58.97 (22.61)	60.80 (20.63)	45.30 (15.87)	68.47 (15.86)
Cupcake_3	57.77 (25.15)	65.27 (19.43)	50.73 (22.78)	48.83 (26.95)	42.83 (16.52)	64.70 (21.57)
Cupcake_4	71.83 (19.34)	67.90 (17.69)	64.13 (23.40)	68.80 (20.46)	46.70 (18.40)	70.60 (17.17)
Donut	68.60 (17.71)	62.83 (20.90)	64.13 (23.40)	63.83 (16.53)	49.25 (12.85)	69.89 (15.20)
Donut_1	71.70 (16.83)	60.27 (22.88)	66.23 (22.05)	61.97 (19.27)	49.10 (13.01)	68.57 (21.36)
Donut_2	60.53 (20.99)	60.60 (24.08)	55.20 (24.84)	54.30 (24.87)	48.00 (14.41)	66.20 (17.07)
Donut_3	76.23 (19.57)	68.97 (23.91)	64.33 (24.23)	76.63 (17.42)	49.40 (18.00)	74.33 (16.33)
Donut_4	65.93 (22.85)	61.47 (21.94)	56.57 (24.57)	62.40 (21.57)	50.50 (16.90)	70.47 (17.73)
Pizza	67.01 (18.36)	61.01 (20.08)	60.40 (21.21)	53.87 (24.76)	71.08 (17.52)	47.11 (27.45)
Pizza_1	67.67 (21.28)	62.90 (23.19)	58.53 (26.45)	50.43 (27.97)	71.70 (17.73)	47.00 (28.57)
Pizza_2	69.53 (21.01)	61.37 (21.14)	62.23 (22.29)	58.77 (28.46)	71.47 (69.93)	45.97 (28.60)
Pizza_3	63.00 (22.14)	58.13 (25.70)	58.80 (23.20)	49.93 (28.51)	69.93 (18.20)	47.27 (28.94)
Pizza_4	67.83 (19.80)	61.63 (20.91)	62.03 (20.79)	56.33 (26.68)	71.23 (18.40)	48.20 (27.47)
Calculator	49.78 (19.22)	46.71 (16.77)	43.20 (20.42)	46.99 (18.73)	40.46 (15.65)	56.17 (20.46)
Calculator_1	51.00 (21.86)	43.77 (23.01)	42.70 (23.39)	47.87 (22.87)	39.60 (16.99)	53.67 (21.82)
Calculator_2	43.67 (25.11)	50.43 (24.36)	43.33 (27.67)	42.73 (29.42)	43.67 (17.71)	56.77 (25.59)
Calculator_3	53.30 (18.31)	49.97 (17.71)	47.13 (19.25)	54.73 (22.17)	40.03 (16.83)	57.13 (20.98)
Calculator _4	51.13 (20.98)	42.67 (18.17)	39.63 (20.71)	42.63 (22.49)	38.53 (15.56)	57.10 (20.31)
Folder	51.48 (15.01)	38.27 (17.50)	39.53 (18.53)	47.53 (14.20)	75.69 (18.77)	55.48 (21.05)
Folder_1	48.57 (20.60)	34.23 (22.61)	33.97 (19.31)	36.90 (24.46)	76.63 (20.12)	52.33 (24.49)
Folder_2	46.30 (23.75)	44.97 (24.27)	39.20 (24.98)	40.20 (27.17)	74.63 (19.04)	57.40 (24.11)
Folder_3	54.00 (16.41)	39.00 (20.20)	43.13 (18.48)	55.77 (16.80)	75.53 (18.36)	55.40 (22.34)
Folder_4	57.07 (19.22)	34.87 (21.20)	41.83 (24.37)	57.23 (21.28)	75.97 (20.10)	56.80 (20.48)
Hole-puncher	51.11 (14.33)	41.86 (16.33)	40.39 (16.65)	48.33 (17.78)	62.38 (16.09)	56.74 (21.64)
Hole-puncher_1	49.63 (17.67)	33.63 (20.80)	37.37 (19.20)	45.17 (22.26)	62.90 (18.62)	58.30 (22.60)
Hole-puncher_2	51.40 (22.06)	45.10 (22.39)	40.87 (22.08)	47.70 (27.99)	61.97 (16.83)	54.13 (26.10)
Hole_puncher_3	56.07 (20.78)	48.63 (20.27)	43.60 (19.94)	57.93 (24.77)	62.00 (15.95)	60.20 (22.36)
Hole_puncher_4	47.33 (17.09)	40.07 (17.83)	39.73 (15.83)	42.53 (21.90)	62.63 (16.96)	54.33 (23.65)
Stapler	52.80 (15.08)	41.16 (20.50)	40.78 (18.29)	52.03 (15.94)	40.38 (19.69)	56.08 (20.93)
Stapler_1	53.73 (15.79)	39.80 (20.58)	36.67 (20.32)	51.33 (20.67)	41.40 (21.72)	54.00 (22.37)
Stapler_2	53.50 (17.62)	42.57 (23.34)	42.57 (20.97)	53.47 (23.72)	40.20 (19.15)	55.20 (21.35)
Stapler_3	50.80 (20.56)	41.97 (24.34)	41.30 (21.62)	47.20 (26.38)	39.23 (19.46)	57.20 (24.33)
Stapler_4	53.17 (17.34)	40.30 (21.89)	42.60 (23.16)	56.10 (20.27)	40.70 (21.50)	57.90 (22.42)

Ratings on VR stimuli were reported on a visual analogue scale ranging from 0 to 100. 100 is reflecting a high score on the corresponding scale, whereas 0 is reflecting a low score. Mean ratings and standard deviations per category and per item are reported.

## Appendix B Ratings of photorealistic stimuli

**Table:**

Stimulus	Mean valence (SD)	Mean arousal (SD)	Mean urge to grasp (SD)	Mean aesthetics (SD)	Mean subjective size (SD)	Mean grasp comfort (SD)
Baseball	58.87 (14.65)	50.87 (18.25)	44.03 (17.87)	53.25 (14.87)	47.87 (18.26)	62.85 (18.46)
Baseball_1	63.60 (16.19)	55.07 (18.41)	48.07 (16.88)	62.13 (20.69)	44.27 (16.43)	65.53 (17.30)
Baseball_2	56.67 (18.99)	51.33 (20.69)	42.27 (19.81)	44.33 (19.26)	51.73 (19.22)	61.73 (18.61)
Baseball_3	52.27 (16.52)	50.60 (20.53)	45.27 (19.56)	47.87 (21.71)	41.53 (18.59)	56.87 (20.72)
Baseball_4	62.93 (22.55)	46.47 (23.76)	40.53 (25.70)	58.67 (25.35)	53.93 (20.31)	67.27 (22.93)
Beachball	58.23 (15.06)	49.32 (20.64)	49.60 (18.99)	55.92 (17.68)	61.98 (16.04)	64.20 (16.73)
Beachball_1	56.27 (21.28)	41.67 (22.54)	48.80 (24.14)	51.93 (18.61)	67.47 (19.57)	63.87 (18.24)
Beachball_2	52.80 (15.62)	60.40 (20.85)	48.93 (20.91)	50.40 (24.18)	65.60 (16.76)	64.20 (20.13)
Beachball_3	67.87 (20.11)	53.73 (23.60)	56.20 (16.06)	67.80 (19.30)	55.13 (17.15)	66.00 (16.72)
Beachball_4	56.00 (8.47)	41.47 (19.32)	44.47 (20.35)	53.53 (13.78)	59.73 (12.90)	62.73 (16.41)
Handball	60.10 (20.43)	57.40 (18.68)	53.27 (22.49)	54.28 (22.03)	61.53 (15.38)	67.03 (16.85)
Handball_1	69.20 (20.95)	53.53 (21.81)	57.33 (25.74)	61.67 (24.67)	58.13 (15.97)	69.67 (15.59)
Handball_2	65.53 (25.28)	60.47 (23.35)	52.00 (28.40)	58.80 (27.49)	66.27 (15.53)	70.53 (20.79)
Handball_3	57.67 (21.28)	64.73 (14.00)	59.07 (18.83)	52.40 (24.96)	60.40 (17.72)	67.00 (17.16)
Handball_4	48.00 (15.31)	50.87 (16.24)	44.67 (17.81)	44.27 (15.55)	61.33 (14.19)	60.93 (15.98)
Tennisball	57.80 (18.41)	52.83 (20.19)	47.78 (21.00)	54.77 (19.82)	48.72 (18.38)	62.27 (18.72)
Tennisball_1	52.87 (16.59)	47.67 (24.25)	46.13 (19.95)	52.27 (18.63)	51.93 (14.86)	60.93 (16.15)
Tennisball_2	61.93 (16.10)	49.47 (23.33)	47.07 (18.18)	58.47 (15.94)	44.73 (17.42)	63.20 (20.44)
Tennisball_3	54.73 (20.79)	53.00 (22.89)	41.20 (22.07)	50.67 (22.60)	52.13 (20.14)	58.47 (20.33)
Tennisball_4	61.67 (23.88)	61.20 (20.91)	56.73 (25.73)	57.67 (31.25)	46.07 (21.67)	66.47 (23.15)
Burger	73.40 (15.05)	68.23 (17.15)	66.25 (21.90)	70.58 (17.16)	59.93 (14.24)	68.78 (15.98)
Burger_1	73.60 (13.03)	66.40 (20.63)	70.07 (16.88)	70.53 (19.11)	56.87 (11.88)	67.80 (11.68)
Burger_2	82.40 (13.45)	71.60 (18.50)	67.00 (24.52)	77.27 (18.09)	59.40 (14.09)	72.07 (14.96)
Burger_3	74.87 (15.02)	63.27 (16.78)	60.13 (23.84)	73.20 (11.27)	58.33 (15.97)	67.67 (16.11)
Burger_4	62.73 (25.06)	71.67 (20.81)	67.80 (25.76)	61.33 (25.47)	65.13 (19.30)	67.60 (25.67)
Cupcake	69.07 (16.04)	62.95 (19.46)	62.28 (21.20)	66.92 (19.40)	52.58 (16.08)	67.88 (16.11)
Cupcake_1	72.60 (14.50)	62.67 (21.29)	63.07 (18.97)	69.80 (22.05)	52.20 (20.27)	67.60 (17.39)
Cupcake_2	62.40 (24.48)	61.00 (18.67)	61.73 (22.36)	55.73 (24.66)	53.40 (17.88)	63.00 (19.73)
Cupcake_3	74.93 (13.83)	66.33 (17.59)	66.87 (23.56)	76.67 (19.63)	49.93 (13.31)	72.40 (14.33)
Cupcake_4	66.33 (18.36)	61.80 (24.79)	57.47 (26.23)	65.47 (20.71)	54.80 (15.17)	68.53 (16.41)
Donut	65.68 (18.18)	56.65 (19.61)	55.63 (23.05)	64.32 (21.39)	51.57 (17.11)	66.97 (16.42)
Donut_1	65.87 (18.63)	55.07 (17.40)	48.47 (22.05)	61.53 (19.20)	51.40 (20.00)	64.20 (20.41)
Donut_2	62.13 (25.13)	56.20 (22.40)	53.27 (25.28)	61.47 (23.25)	47.47 (18.69)	64.40 (17.53)
Donut_3	73.53 (16.22)	59.33 (21.96)	58.87 (26.99)	75.07 (17.13)	59.87 (18.60)	71.00 (17.62)
Donut_4	61.20 (23.58)	56.00 (25.63)	61.93 (20.76)	59.20 (32.09)	47.53 (15.15)	68.27 (15.49)
Pizza	77.78 (14.85)	70.45 (18.27)	67.92 (22.27)	76.18 (17.44)	64.12 (21.98)	72.78 (14.97)
Pizza_1	77.87 (12.56)	67.67 (18.82)	69.87 (18.76)	77.73 (19.96)	63.80 (23.82)	71.27 (15.40)
Pizza_2	79.13 (14.53)	72.80 (14.79)	71.87 (24.29)	84.20 (13.46)	62.53 (19.87)	75.33 (15.67)
Pizza_3	74.33 (17.27)	65.00 (21.23)	62.60 (19.69)	65.73 (24.07)	65.13 (22.51)	69.87 (15.40)
Pizza_4	79.80 (19.87)	76.33 (22.47)	67.33 (29.48)	77.07 (18.69)	65.00 (24.67)	74.67 (17.85)
Calculator	47.88 (18.81)	37.57 (22.12)	37.22 (20.75)	39.67 (17.38)	46.97 (15.61)	59.35 (21.25)
Calculator_1	44.67 (14.89)	25.40 (15.70)	28.93 (15.48)	31.53 (13.96)	40.87 (18.02)	56.20 (22.31)
Calculator_2	49.33 (23.74)	49.53 (22.68)	43.13 (21.98)	37.87 (26.01)	49.93 (18.34)	56.40 (17.19)
Calculator_3	45.47 (16.23)	40.00 (22.88)	36.40 (20.65)	40.13 (18.27)	44.13 (14.96)	61.33 (23.82)
Calculator _4	52.07 (22.74)	35.33 (27.82)	40.40 (25.94)	49.13 (18.10)	52.93 (18.99)	63.47 (26.37)
Folder	52.10 (15.35)	37.37 (22.31)	37.68 (20.27)	50.83 (16.76)	64.65 (21.46)	65.50 (19.77)
Folder_1	47.40 (19.57)	30.73 (29.16)	28.20 (21.01)	41.27 (20.12)	63.73 (23.28)	67.33 (20.87)
Folder_2	47.73 (18.66)	42.60 (25.96)	39.00 (25.28)	47.13 (27.15)	65.67 (23.12)	59.47 (24.75)
Folder_3	54.13 (13.75)	39.20 (22.59)	40.47 (24.72)	56.67 (11.51)	64.27 (23.79)	70.67 (18.83)
Folder_4	59.13 (16.75)	36.93 (20.58)	43.07 (15.70)	58.27 (18.87)	64.93 (19.20)	64.53 (17.71)
Hole-puncher	54.53 (14.24)	34.48 (21.13)	38.88 (15.81)	49.38 (17.11)	50.38 (14.92)	62.43 (17.67)
Hole-puncher_1	54.53 (13.38)	34.87 (27.19)	35.47 (20.12)	49.40 (24.35)	48.27 (17.16)	62.53 (20.63)
Hole-puncher_2	53.87 (20.06)	37.20 (22.28)	40.13 (21.75)	49.60 (21.37)	57.73 (16.88)	62.33 (20.65)
Hole_puncher_3	50.53 (19.19)	34.53 (20.42)	41.00 (14.29)	46.80 (16.35)	45.93 (14.39)	61.07 (14.84)
Hole_puncher_4	59.20 (13.63)	31.33 (22.48)	38.93 (17.60)	51.73 (18.02)	49.60 (12.98)	63.80 (19.21)
Stapler	50.08 (14.84)	36.68 (22.77)	37.53 (19.96)	44.12 (18.17)	46.12 (18.00)	63.68 (19.64)
Stapler_1	52.40 (14.81)	32.73 (25.27)	29.47 (19.63)	44.93 (17.66)	39.67 (15.89)	63.60 (20.65)
Stapler_2	51.33 (18.23)	32.33 (22.97)	40.93 (23.48)	47.07 (19.52)	52.87 (19.22)	69.67 (20.58)
Stapler_3	46.00 (17.01)	37.00 (25.88)	32.87 (18.57)	33.93 (26.34)	41.13 (14.99)	55.60 (21.41)
Stapler_4	50.60 (17.26)	44.67 (20.73)	46.87 (19.15)	50.53 (19.44)	50.80 (21.00)	65.87. (17.26)

Ratings on photorealistic stimuli were reported on a visual analogue scale ranging from 0 to 100. 100 is reflecting a high score on the corresponding scale, whereas 0 is reflecting a low score. Mean ratings and standard deviations per category and per item are reported

## Appendix C FCQ-S before and after the behavioural task in the VR

**Table:**

Scale	Mean score pre (SD)	Mean score post (SD)	*t* test
Desire to eat/loss of control	13.23(4.04)	14.07(5.23)	*t*(29) = 1.21, *p* = 0.236
Positive affect	15.03(4.61)	15.93(5.66)	*t*(29) = 1.72, *p* = 0.096
Hunger	9.2(2.66)	10.20(2.61)	*t*(29) = 3.94, *p* < 0.001

The FCQ-S assesses the desire to eat a specific food as a state variable. 15 items subdivide into three subscales: intense desire to eat/loss of control (6 items), positive affect (6 items) and hunger (3 items). Each item scores from 1 (“strongly disagree”) to 5 (“strongly agree”). Mean ratings and standard deviation for each scale are depicted. *t* Tests for dependent samples were performed to estimate pre-post-differences

## Appendix D Reaction times without standardization

**Table:**

	Mean RT balls (SD)		Mean RT food (SD)		Mean RT office tools (SD)	
	VR	Touchscreen	VR	Touchscreen	VR	Touchscreen
Movement onset	521.92 (99.49)	455.05 (81.49)	574.33 (103.86)	470.69 (85.67)	590.89 (114.69)	491.09 (85.18)
Collection time	1049.01 (343.89)	766.82 (171.83)	1139.03 (397.71)	797.86 (180.39)	1089.68 (367.05)	790.21 (174.24)

The table depicts raw mean reaction times and standard deviations of the movement onset and collection time for each stimulus category (balls, food, office tools) dependent on the medium (VR, touchscreen)

## Appendix E fNIRS-BOLD response of ball-standardized food- and office tool-condition

**Table:**

Brodmann area (BA)	L ch	*t*	*p*_cor_	R ch	*t*	*p*_cor_
Ball-standardized food vs. office tool condition						
BA 9	8	1.76	1	13	0.80	1
	11	1.12	1	15	1.14	1
				16	0.28	1
				18	0.61	1
				20	0.23	1
BA 44	6	1.29	1	23	0.37	1
BA 45	2	− 2.44	0.376	24	− 0.41	1
	4	1.24	1			
	7	2.59	0.268			
BA 46	5	− 1.05	1	19	3.29	0.047*
	9	0.48	1	21	0.66	1
				22	2.18	0.673

* Indicates *p* < 0.05. ** indicates *p* < 0.01. Reported *p* values were Bonferroni-corrected for multiple comparisons.
